# “The only way that they can access help quickly”: a qualitative exploration of key stakeholders’ perspectives on guided self-help interventions for children and young people with eating disorders

**DOI:** 10.1186/s40337-024-01113-w

**Published:** 2024-09-30

**Authors:** Emily Davey, Nadia Micali, Rachel Bryant-Waugh, Sophie D Bennett, Charmayne Lau, Roz Shafran

**Affiliations:** 1 UCL Great Ormond Street Institute of Child Health, University College London, 30 Guilford Street, London, WC1N 1EH UK; 2 Center for Eating and Feeding Disorders Research (CEDaR), Mental Health Services of the Capital Region of Denmark, Ballerup Psychiatric Centre, Copenhagen, Denmark; 3 Maudsley Centre for Child and Adolescent Eating Disorders, South London and Maudsley NHS Foundation Trust, London, UK; 4 Department of Child and Adolescent Psychiatry, Institute of Psychiatry, Psychology & Neuroscience, King’s College London, London, UK; 5 Department of Psychology, Institute of Psychiatry, Psychology & Neuroscience, King’s College, London, UK

**Keywords:** Eating disorders, Children, Young people, Guided self-help, Low intensity psychological intervention, Intervention development, Qualitative study

## Abstract

**Background:**

There is a significant unmet treatment need for children and young people (CYP) with eating disorders. Guided self-help interventions have the potential to expand access to evidence-based treatments. Guided self-help is a type of low intensity psychological intervention where individuals engage with a workbook or online programme, with the support of a health professional. Its primary aim is to equip patients and/or their caregivers with self-management skills. However, little is currently known about the acceptability and suitability of guided self-help interventions for CYP with eating disorders. This study aimed to explore the perspectives of three key stakeholder groups – CYP with lived experience of eating disorders, parents/carers, and healthcare professionals – on guided self-help for this population.

**Methods:**

Qualitative focus groups and semi-structured interviews were conducted with 11 CYP (aged 13–19 years) with lived experience of eating disorders, 12 parents/carers, and 10 healthcare professionals. The study comprised a total of seven focus groups (including 2 with CYP, 3 with parent/carers, and 2 with healthcare professionals), as well as four semi-structured interviews (including 3 with CYP and 1 with a parent/carer). Discussion topics included past experiences of using/delivering guided self-help, the suitability of guided self-help for CYP with eating disorders, and preferences towards the content, structure and modes of guided self-help. Data were analysed using reflexive thematic analysis.

**Results:**

Three themes were generated across all three stakeholder groups. Theme one, *Bridging the gap*, highlighted the role of guided self-help in increasing access to psychological support for CYP with eating disorders. Theme two, *Timing matters*, considered the suitability of guided self-help for CYP with eating disorders at different stages of illness and the care pathway. Theme three, *One size does not fit all*, emphasised the heterogeneity of eating disorders and the need for a personalised and flexible approach in guided self-help.

**Conclusions:**

Findings from this study lay a foundation for the future design and delivery of guided self-help interventions for CYP with eating disorders. Future work must consider these findings in the context of best available research evidence to optimise the potential utility of guided self-help for this population.

**Supplementary Information:**

The online version contains supplementary material available at 10.1186/s40337-024-01113-w.

## Abstract (plain language summary)

Eating disorders are prevalent among children and young people, yet accessing effective treatment often involves significant delays. Guided self-help interventions, which are shorter and require less therapist involvement than standard treatments, could help bridge this gap. However, guided self-help is not readily available for this population. The current study explored the perspectives of three key stakeholder groups – children and young people with lived experience of eating disorders, parents, and healthcare professionals – on guided self-help interventions. Overall, findings suggest that guided self-help interventions are perceived as an acceptable treatment option that holds promise in providing timely access to treatment for children and young people with eating disorders. Future work must take into account these perspectives alongside the best available research evidence to optimise the use of guided self-help for this patient group.

**Supplementary Information:**

The online version contains supplementary material available at 10.1186/s40337-024-01113-w.

## Background

Eating disorders cause detrimental impairment in physical and mental health [1], and have one of the highest mortality rates among psychiatric disorders [2]. The prevalence of eating disorders among children and young people (hereafter referred to as ‘CYP’) has increased substantially in recent years, with the COVID-19 pandemic having a discernible impact on this trend [3, 4]. Recent findings from the Mental Health of Children and Young People (MHCYP) survey in England indicate that 2.6% of CYP aged 11–16, and 12.5% of those aged 17–19, have a diagnosable eating disorder, according to population estimates [5]. This marks a notable increase from the respective rates of 0.5% and 0.8% reported in the 2017 survey [5]. Unsurprisingly, child and adolescent eating disorder services in the UK have struggled to meet this unprecedented demand; many CYP are facing long waits for evidence-based treatment and waiting time standards are regularly being breached [6]. Delays are not without consequences – a long duration of untreated eating disorder (otherwise known as ‘DUED’) can lead to a protracted illness course and poorer health outcomes [7, 8]. This unmet need for treatment among CYP with eating disorders is a pressing concern that necessitates prompt and concerted action. There is an imperative to reconsider how psychological support is delivered to this population, with the aim of increasing access to evidence-based treatment [9].

Self-help interventions present an opportunity to improve the accessibility of evidence-based treatments. These interventions involve individuals engaging with a workbook, app or computer-based programme designed to educate them about their disorder and equip them with self-management skills to overcome their difficulties [10]. Self-help interventions involving direct support from health professionals, known as ‘guided’ self-help, have been shown to achieve better adherence and treatment outcomes compared to ‘pure’ self-help approaches [11]. By delivering evidence-based treatments in a format that demands less clinician time, cost and specialist training to deliver [12, 13], guided self-help interventions hold considerable potential to close the treatment gap for CYP with eating disorders.

The National Institute for Health and Care Excellence (NICE) recommends guided self-help as the first-line treatment for adults with bulimia nervosa and binge eating disorder [14], and there is emerging evidence to suggest that these interventions may be effective for adolescents with bulimia nervosa [15] and anorexia nervosa [16, 17]. However, guided self-help interventions are not yet widespread in their evidence base and availability for CYP with eating disorders.

The successful implementation of an intervention relies on its acceptability to both those receiving the intervention (e.g., patients and caregivers) and those facilitating its delivery (e.g., healthcare professionals and caregivers) [18]. Patients are more likely to adhere to treatment recommendations and ultimately benefit from improved clinical outcomes if they consider an intervention to be acceptable [19, 20]. Treatment acceptability may be influenced by factors such as the appropriateness and suitability of the intervention content, as well as the context and quality of the treatment provided [20, 21]. The perceived acceptability of an intervention among healthcare professionals plays a pivotal role in ensuring its optimal delivery [19, 20].

There has been increasing recognition of qualitative methodologies in the development and optimisation of complex interventions [18]. Qualitative approaches are well suited to gain a comprehensive understanding of user needs and issues relating to the acceptability and feasibility of interventions, as they allow for in-depth exploration of the user perspective and the implementation context [22, 23]. A meta-synthesis of qualitative studies on users’ experiences of self-help interventions for eating disorders across the age range identified several common perceived advantages, including accessibility, convenience, confidentiality and increased autonomy over recovery [24]. Drawbacks included issues with self-motivation and scepticism about treatment effectiveness. The helpfulness of guidance was emphasised across all studies. However, these studies gathered users’ views on specific self-help interventions post-intervention, rather than prior to intervention development. As a result, these data may be positively biased as dissatisfied participants are more likely to become non-completers [25, 26] and not participate in subsequent interviews. The Medical Research Council (MRC) recommends engaging with key stakeholders during the development process to help maximise the potential effectiveness of an intervention, as well as enhance the prospects of policy and practice change [18].

Many of the studies investigating stakeholder perspectives on self-help interventions for eating disorders outside of an intervention study are quantitative in nature [26–28]. Such survey-based designs are less adept at gathering in-depth and detailed information that can be yielded qualitatively. To our knowledge, only two studies have used qualitative methods to explore stakeholders’ attitudes towards self-help for eating disorders prior to an intervention [25, 30]. Their combined findings indicate that online self-help interventions offer benefits such as accessibility, immediate support and overcoming fears of stigmatisation. However, participants identified barriers to initiation (e.g., feeling overwhelmed or isolated, lack of accountability) and engagement (e.g., scientific jargon, excessive effort), along with concerns about data safety. Stakeholders emphasised the need for online self-help interventions that are user-friendly, evidence-based and offer personal support. However, these studies are limited in that Yim and colleagues [30] recruited adults with eating disorders characterised by recurrent binge eating only, and Schmidt-Hantke and colleagues [25] did not report on the age or eating disorder diagnosis of patient participants. Consequently, these studies may not reflect the specific developmental needs of CYP with eating disorders. Furthermore, both studies focused on online self-help formats and neither specified whether their focus was on guided self-help or ‘pure’ self-help. Although there is a common assumption that CYP would favour online interventions due to their ubiquitous digital activity [31], this remains uncertain for CYP with eating disorders [32, 33]. To our knowledge, there is currently no user-centred research on guided self-help interventions that incorporates the perspectives of CYP with eating disorders. Given the potential of guided self-help to increase access to evidence-based treatment for this population [12, 13], further exploration is warranted.

The current study aimed to explore the perspectives of three key stakeholder groups (CYP with lived experience of eating disorders, parents and healthcare professionals) on guided self-help interventions for CYP with eating disorders. Specific objectives were to:

1. Gain insights into stakeholders’ views and attitudes regarding the acceptability of guided self-help interventions for CYP with eating disorders.
2. Understand stakeholders’ preferences for access to and delivery of a guided self-help intervention for CYP with eating disorders.
3. Determine the informational and content requirements for a guided self-help intervention for CYP with eating disorders.

## Methods

This study received ethical approval from the University College London Research Ethics Committee (22129/001) and is reported in accordance with the COnsolidated criteria for Reporting Qualitative Research (COREQ) checklist [34].

### Participants and recruitment

CYP with lived experience of an eating disorder, parents and healthcare professionals were recruited through poster advertisements shared on social media platforms such as Twitter and Facebook, as well as by UK-based eating disorder charities like Beat, and private treatment providers. The poster invited potential participants by asking, ‘Are you a young person or a parent/carer of a young person with an eating disorder? You may be eligible to take part in a focus group to share your views on guided self-help treatments for children and young people with eating disorders’. Adverts directed at healthcare professionals were similar but posed the question, ‘Are you a healthcare professional working with children and young people with eating disorders?’. Study adverts included a link to an online information sheet, which invited those interested to complete a consent to be contacted form or to email the research team to express their interest. The lead researcher (*removed for peer review*) contacted those interested via telephone/email to discuss the study and to seek informed consent for their participation. Written informed consent was obtained from all participants, and from parents when participants were under 16 years old.

All participants completed a screening questionnaire to determine their eligibility to take part. For CYP and parents, this included demographic information and information about the child’s mental health diagnoses and their history of psychological support. For healthcare professionals, this included demographic information and details about their clinical work (e.g., their work setting, years of experience).

CYP were eligible to take part if they were 11–19 years old and self-reported a current, or previous, diagnosis of anorexia nervosa, bulimia nervosa, binge eating disorder or other specified feeding or eating disorder (OSFED). The selection of this age range was informed by the elevated population prevalence estimates of eating disorders among CYP in England, with 2.6% of CYP aged 11–16 and 12.5% of those aged 17–19 affected [5]. Parents were eligible to take part if they were a parent/caregiver of a child who met this eligibility criteria, or if they had a child (aged up to 19 years) with a lifetime diagnosis of avoidant/restrictive food intake disorder (ARFID). Healthcare professionals were eligible to take part if they had more than one year experience in treating CYP with eating disorders or other common mental health conditions (e.g., anxiety and depression). Participants were excluded only if they did not have sufficient English language skills to participate in a focus group or interview.

The principles of information power guided recruitment and data collection; sample size was determined by the amount of information needed to adequately address the research question [35]. Recruitment continued until analyses indicated that the dataset was sufficiently rich and varied to address study aims [36]. CYP and parents received a £40 gift voucher as compensation for their time.

### Procedure

#### Data collection

Participants took part in a focus group discussion or a semi-structured interview with the first author (*removed for peer review*), depending on their preference and availability. Separate focus groups were conducted with each set of stakeholders to avoid a perceived hierarchy among mixed patient and clinician groups. All focus groups and interviews were conducted via Zoom.

At the outset of each focus group and interview, the lead researcher delivered a brief presentation to participants to establish a baseline understanding of guided self-help. This presentation covered what guided self-help is, what its used for and the study rationale (i.e., to gather perspectives on guided self-help interventions for CYP with eating disorders to inform future intervention development). The researcher ensured that participants understood the concept of guided self-help before proceeding with the interview questions.

Focus groups and interviews followed a flexible topic guide, developed based on relevant literature (e.g., 22,27) and the research team’s experience of working with CYP with eating disorders. The topic guides were tailored accordingly for each stakeholder group, but all covered: personal experiences of using/delivering guided self-help; suitability of guided self-help for CYP with eating disorders; preferences towards the content, structure and modes of guided self-help (see Additional File 2). The healthcare professional topic guide was piloted prior to data collection to ensure clarity of questions and that relevant data were collected. Field notes were taken during and after each focus group and interview discussion.

Focus groups averaged 85 min (range 54–107 min) and interviews averaged 52 min (range 45–65 min). Interviews were audio-recorded and transcribed verbatim, with identifiable information redacted and random participant identification numbers assigned.

### Data analysis

Reflexive thematic analysis was used to identify common patterns and meanings across the dataset [37, 38]. The dataset was analysed inductively (directed by the content of the data) and semantically (reflecting the explicit content of the data). Emerging findings were shared and discussed at regular supervisory meetings to allow for alternative interpretations of the data. (*Removed for peer review*) met weekly with her primary supervisor (*removed for peer review*), and monthly with her subsidiary supervisors (*removed for peer review*) throughout data analyses. A joint analysis of the results of the three stakeholder groups was considered more informative for the future development of a guided self-help intervention for CYP with eating disorders. Consequently, distinctions between groups are drawn solely in instances when their perspectives contradicted each other. *NVivo* was used to support data analysis and organisation.

During analyses, it was evident that several themes pertained more closely to stakeholders’ overarching views on guided self-help interventions and their suitability for CYP with eating disorders, while others delved into more specific topics and features that should be incorporated into the intervention. Only the themes relating to the aims of the study in relation to stakeholders’ broad perspectives on guided self-help interventions for CYP with eating disorders are reported here.

## Results

### Participant characteristics

Forty-six individuals (16 CYP, 18 parents/carers, and 12 HCPs) expressed an initial interest in the study, and 33 participated in a focus group or interview (71%). Participants included 11 CYP with lived experience of eating disorders, 12 parents/carers, and 10 healthcare professionals. In total, seven focus groups (2 CYP, 3 parent, 2 HCP) and four semi-structured interviews (3 CYP, 1 parent) were conducted. Each focus group contained between 3 and 6 participants. Please refer to Tables 1, 2 and 3 in Additional File 3 for an overview of which participants were present within each focus group and interview.

CYP ranged in age from 13 to 19 years (*M* = 15.45; SD = 2.16). They were majority female (*N* = 10; 91%) and of White British ethnicity (*n* = 10; 91%). Eight CYP reported a current diagnosis of anorexia nervosa, two individuals reported having OSFED, and one young person reported having lived experience of both anorexia nervosa and bulimia nervosa. Eight CYP were currently undergoing treatment for their eating disorder, either through the NHS, private sector or third sector organisations, while the remaining two had previously received treatment. None of the CYP had used any form of self-help intervention for their eating disorder; however, one CYP disclosed prior use of guided self-help for anxiety.

Parents were recruited at the same time as CYP; however, not all dyads opted to take part, resulting in some parents participating without their child and vice versa. All parents were mothers of CYP with lived experience of either anorexia nervosa (*n* = 9) or OSFED (*n* = 3). Six were mothers of CYP who also participated in the study. Their average age was 49.25 years (range = 42–55 years; SD = 4.31) and they were majority White British (*n* = 9; 83%). Three parents reported current or prior use of the book ‘Skills-based caring for a Loved One with an Eating Disorder: The New Maudsley Method’ [39], delivered as a guided self-help intervention by a third sector organisation.

All participating healthcare professionals had experience of treating CYP with eating disorders. Their average age was 38.11 years (range = 24–52 years; SD = 8.43), most were female (*n* = 7; 77.8%) and of White British ethnicity (*n* = 9; 90%); one healthcare professional preferred not to disclose their age or gender. Healthcare professionals’ occupations included: Clinical Psychologist (*n* = 4), Mental Health Nurse (*n* = 3), Consultant Psychiatrist (*n* = 1), Assistant Psychologist (*n* = 1), CBT Therapist (*n* = 1), Family Therapist (*n* = 1), and Clinical Support Worker (*n* = 1). Two healthcare professionals identified with two occupations; hence the total of these numbers exceed the healthcare professional sample size of 10. In the presentation of these results, CYP will be identified as (CYP), parents as (P) and healthcare professionals as (HCP). See Tables 1, 2 and 3 in Additional File 3 for more details.

**Fig. 1:**
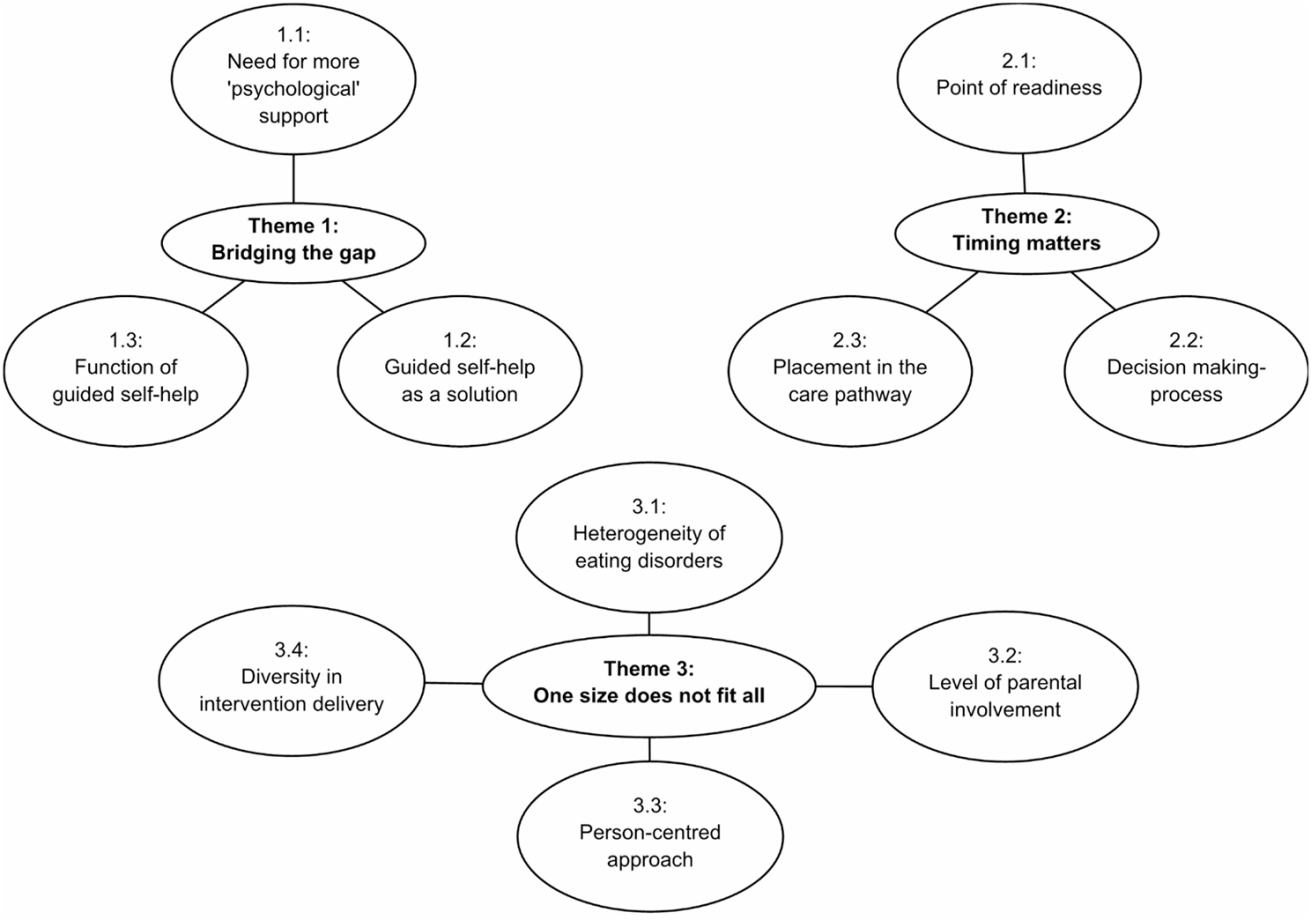
Thematic map

Three overarching themes were generated from the data: bridging the gap, timing matters and one size does not fit all (see Fig. 1 for a thematic map). These themes are described in detail below and evidenced by key quotations embedded within the text.

## Theme 1: Bridging the gap

In the first theme, stakeholders discussed the unmet mental health needs among CYP with eating disorders and the role of guided self-help in bridging this treatment gap.

### Need for more ‘psychological’ support: “there’s just too much focus on weight” (CYP6)

Recounting their previous treatment experience, both CYP and parents conveyed their perception that existing treatment for eating disorders lacks a psychological focus. Many conceptualised eating disorders as a ‘coping strategy’ and emphasised the necessity of developing alternative ways to cope with their thoughts and feelings. However, participants felt that there was too much emphasis on physical recovery and weight restoration and not enough psychological support for CYP.

*CYP4: “I just wanted to focus on how to actually get over it […]*,* I felt like a lot of the time*,* a lot of the focus was put on how to recover physically rather than like mentally”.*

This perspective was corroborated by a healthcare professional whose clinical experience indicated that current treatment may not consistently align with families’ preconceived notions of traditional therapy:

*HCP6: “I think that one of the complications in AN treatment is that […] sometimes young people and parents feel like it’s not*,* um it’s not very psychological. It doesn’t necessarily look like what they think therapy looks like. So*,* I’ve known parents to be like ‘so when does the therapy start?’”*.

### Guided self-help as a solution: “It’s vital to have this sort of thing” (P11)

Stakeholders identified guided self-help interventions as a promising solution to address the existing gap in psychological support for CYP with eating disorders. Both parents and healthcare professionals highlighted the inadequacy of current service provision due to a lack of available resources. They considered that guided self-help may be the only viable option for increasing access to psychological treatment:

*P11: “There’s no way*,* unless we have a huge influx of funding*,* there’s no way that services can keep up with what’s required and what’s needed*,* and therefore self-help is gunna be the only way that they can access help quickly”*.

Stakeholders emphasised that self-help resources tend to encompass more *“psychological concepts and thinking” (HCP6)* and that guided self-help may offer a nuanced approach to help CYP manage their disordered eating thoughts and feelings.

*(P6): “I think that for children*,* it’s knowing how to manage those feelings isn’t it? The feelings*,* the emotions […] and I think if they could have some kind of self-help book*,* I think that would be really useful”*.

However, there was apprehension among participants regarding the adult-centric nature of existing self-help interventions for eating disorders. Concerns were raised about available resources being geared towards topics that are often irrelevant and unfamiliar to CYP:

*HCP1: “It’s the same for lots of self-help material that’s out there*,* it’s so adult-focused in terms of […] talking about transitions and retirement and kind of mortgages and things which are just so alien to so many young people”*.

### Function of guided self-help: “I think it’s just having that toolkit to manage those feelings” (P6)

Participants highlighted several potential benefits of guided self-help interventions for CYP with eating disorders. They acknowledged the value of guided self-help in providing psychoeducation and enhancing knowledge about eating disorders. CYP spoke about the role of guided self-help in fostering self-discovery and empowering individuals to gain insight into their own experiences: *“Obviously it will give them more knowledge around like their struggles and give them like almost clarity” (CYP11); “For me it’s about like finding out more about myself and trying to put things together” (CYP14).* CYP and parents stressed the supportive role of a guide in facilitating a better understanding of the self-help material and to help clarify aspects that are unclear: *“I think it’s also quite hard when you don’t have a professional there because you can’t… if you don’t understand something or if you’re unsure*,* you can’t do anything about that” (CYP11).*

Stakeholders emphasised the need for guided self-help to be a trustworthy and credible resource amidst the abundance of conflicting information on the internet: *“It’s that reference point to go back to and go ‘you know this is actually true like I’m not just reading this off the internet” (CYP10).* They stressed the importance of ensuring that guided self-help interventions are grounded in clinical evidence. One healthcare professional, *HCP4*, highlighted the necessity for evidence-based information: *“You know*,* what you’re delivering […]*,* the information that they’re getting is based on evidence you know*,* it’s not just information that clinicians have plucked”.* Similarly, parents spoke about how the intervention being affiliated with the NHS and endorsed by healthcare professionals is a crucial aspect for establishing credibility and trust: *“It needs to say that it is NHS-based and you know*,* that it’s supported by doctors and medical professionals. I think that’s the key thing” (P1).*

The majority of participants expressed the view that a primary goal of self-help interventions is to equip CYP with “*different techniques to help them cope” (CYP9)*. They advocated for a behavioural focus, emphasising the need for practical tools within self-help materials. Both CYP and parents felt that guided self-help should empower CYP to engage in alternative coping strategies instead of disordered eating behaviours: *“I suppose with the young people*,* it’s when they’re getting all these horrible thoughts and voices […]*,* it’s knowing for them what they can do*,* the distraction techniques’” (P6).* A few CYP highlighted that it can be challenging to independently develop strategies from information alone. They explained that a guide can help facilitate this process by prompting the individual to consider how the strategies can be applied and integrated into their own life.

*CYP10: “It’s quite hard sometimes to make strategies out of information*,* but if you’re given them or given ideas to get you thinking about how that can be implemented and brought into your life”.*

The role of a guide in sustaining motivation throughout the self-help intervention was also emphasised: *“It would be more difficult to have the motivation to complete the help by yourself*,* but if you had a professional there*,* you have like constant motivation to actually continue because there’s someone supporting you whilst you’re doing it” (CYP2).*

## Theme 2: Timing matters

In the second theme, participants gave their different perspectives on the pivotal role of timing within the context of guided self-help interventions for CYP with eating disorders.

### Point of readiness: “In order to be receptive to any kind of psychological or psychiatric input, you need to be at a certain place” (P9)

There was consensus among stakeholders that having a certain level of readiness or receptivity is crucial for meaningful engagement with guided self-help interventions. Participants shared the view that CYP must reach a distinct cognitive or emotional state, often referred to as a *“certain place”* (P9), before they can effectively engage with and derive benefit from such interventions. The inherently subjective nature of this ‘point of readiness’ was emphasised, with many participants recognising that there is no set timeframe for reaching this state.

*CYP5: “I do think that there is kind of a stage where it would be helpful*,* and also where it wouldn’t really. But I guess that’s really*,* you know*,* individualistic”*.

Referring to CYP with anorexia nervosa, stakeholders discussed the impact of malnutrition on cognitive function and noted how being severely underweight can hinder a person’s ability to comprehend information and apply concepts to their everyday life. One parent, *P2*, expressed this concern by stating: *“At the lowest weight*,* you know when the brain is absolutely starving*,* they can’t really take anything on board”.* Consequently, stakeholders were of the opinion that *“weight restoration is the key factor initially that you need” (HCP10)* before a young person who is severely underweight is able to engage with guided self-help. However, they highlighted a crucial dilemma: the challenge of motivating CYP to alter their eating habits sufficiently for weight gain such that they reach a ‘point of readiness’.

*P2: “Not when they’re starving because they just can’t engage… perhaps when they’re starting to get a bit more weight restored. But it’s a chicken and egg thing isn’t it. How do you get them to eat so they’re weight restored so that they can engage?””*.

Participants emphasised the importance of internal motivation for a young person to actively participate in guided self-help and highlighted that sustained and meaningful change comes from the individual’s own desire and willingness to embrace change.

*CYP5: “I mean there is a part in recovery where*,* you know*,* all these professionals they force it on you*,* they force you to eat*,* they force to you to do this*,* do that*,* to follow all these rules*,* but I think that at the end of the day*,* nothing will change unless you want that change”*.

*P5: “I think for me it’s when you’ve got those green shoots*,* where they’re saying ‘actually Mum*,* I want to get better*,* I’ve had enough of this’. I think that’s a really good point then isn’t it to go ‘right’… but I think when they’re still fighting with you on that*,* then it’s […] kind of hard”*.

Some healthcare professionals suggested that CYP with eating disorders characterised by recurrent binge eating may have more motivation to engage with guided self-help than those with anorexia nervosa: *“It’s not obviously strictly true for everybody that we meet*,* but […] I feel like there’s probably a lot more motivation for more of a self-help approach*,* um*,* in binge eating*,* or maybe even like bulimia presentations compared to maybe the presentations that we see in young people struggling with anorexia” (HCP9).*

### Decision-making process: “It’s about risk” (HCP5)

Healthcare professionals highlighted the importance of risk assessment in the decision-making process when considering guided self-help for CYP with eating disorders. There was consensus that such interventions should not be offered to CYP who present with high risk. Healthcare professionals spoke about their reluctance to recommend guided self-help to CYP in an outpatient setting unless the perceived risk was low. One healthcare professional, *HCP10*, specified exclusion criteria in relation to risk: *“People with extremely low weight or people who are rapidly losing weight*,* that would be an exclusion criteria for us*,* alongside really low mood”*.

Beyond these criteria, healthcare professionals expressed challenges in making a priori decisions about who would respond well to guided self-help, describing it as a *“leap of faith” (HCP10).* One healthcare professional, *HCP1*, reflected on their experience of working with families with family-based therapy (FBT): *“I really can’t guess who’s going to do well with FBT when we deliver it to start with*,* and sometimes I’m shocked that the parents I think are going to be ones that are going to roll with this model*,* don’t. So […] this has knocked my confidence in terms of differentiating who’s going to be appropriate for a kind of more low intensity*,* kind of intervention than others”*.

### Placement in the care pathway: “It really is about being offered as a treatment in and of itself” (HCP9)

There were discussions among stakeholders about the optimal placement of self-help interventions in the care pathway for CYP with eating disorders. Many stakeholders advocated for guided self-help as an early intervention to prevent the escalation and worsening of the eating disorder: “*I think the longer you leave it the stronger the eating disorder gets” (CYP9); “I think it’s easier to implement coping strategies and like ways to get out of getting too far into your illness when it’s early” (CYP11).* A minority of healthcare professionals viewed self-help as the first step in a stepped-care model, describing it as a *“holding intervention” (HCP6)* and as a prelude to getting *“more formal*,* structured form of therapy” (HCP4).*

Stakeholders also considered the role of guided self-help as an adjunct to therapist-delivered care. Some healthcare professionals expressed the view that self-help interventions could complement and enhance the effectiveness of individual therapy. Notably, a healthcare professional working in an inpatient service recognised the potential benefit of self-help for CYP in such settings: *“Although you wouldn’t want the young person to sort of fix their eating disorder by themselves indefinitely*,* the ability to at least do something on their own steam with some kind of framework or guidance would be helpful” (HCP3).*

Healthcare professionals additionally suggested guided self-help as a potential tool for relapse prevention, serving as a resource to reinforce learned techniques and maintain progress: *“I’m also thinking about like if someone’s had an intervention*,* has been doing quite well*,* but has maybe a bit of a lapse*,* it might be really useful at that point as well*,* just to kind of sort of remind them of the techniques… to kind of pull on those resources that they’ve got*,* almost like a bit of a top up” (HCP9).*

Irrespective of its placement in the care pathway, the majority of healthcare professionals felt strongly that guided self-help should not be viewed merely as a *“stopgap” (HCP9)*, asserting its potential as an effective treatment for CYP with eating disorders. One participant, *HCP5*, stressed the need to accord the intervention the same level of importance as other forms of psychological support, stating *“It needs to be given as much kudos as anything else”*. There was consensus among stakeholders that there needs to be a robust system or “scaffolding” *(HCP10)* from services to support the delivery of guided self-help. This service structure would outline processes for escalating risk, discontinuation of treatment and referral to more intensive interventions: *“It doesn’t really matter about [the guide’s] kind of job title as it’s more about the support they’ve got in order to be able to offer [guided self-help] and um feeling supported… and there’s a structure in place for*,* you know*,* things like escalating risk*,* or whatever it might be*,* or the intervention doesn’t seem like it’s meeting the needs” (HCP2).*

## Theme 3: One size does not fit all

The third theme acknowledges the diverse nature of CYP with eating disorders and highlights the necessity for a personalised and flexible guided self-help intervention for this population.

### Heterogeneity of eating disorders: “Every child is unique in what’s going on for them” (P7)

Stakeholders reflected on the heterogeneous nature of eating disorders, acknowledging the uniqueness of each child’s experience of their eating disorder and the variability in the underlying causes and presentations of the condition: *“I think we’ve all agreed that every child is so different. The triggers of why they started were different and how you help the recovery is different”* (*P1).* A recurring topic throughout the discussions with all three stakeholder groups was the high incidence of comorbidities among eating disorders, particularly anxiety, depression, and autism spectrum disorder. CYP and parents spoke about the challenges of managing these co-occurring conditions in the context of an eating disorder: *“When you have loads of different things like anxiety and the eating disorder*,* it kind of like gets in the way” (CYP7).* They shared the perspective that integrating strategies and content that addresses these co-occurring conditions could enhance the acceptability and effectiveness of the guided self-help intervention: *“It’s really difficult for me cause I have to suffer with depression and anxiety so maybe include how to like deal with those kind of things*,* cause once you kind of know how to deal with those things*,* it’s like kind of easier to deal with an eating disorder” (CYP6).*

### Level of parental involvement: “You want to be independent at a certain point” (CYP10)

The level of parent involvement in the guided self-help intervention was seen as dependent on the age and autonomy preferences of the CYP. The collective perspective from stakeholders suggests that younger children may benefit from more parental involvement, while older adolescents may prefer increased autonomy: *“I think when they’re young they definitely need their parents help. But if they’re like late teenagers*,* they might not want their parents involved” (CYP9).* Participants acknowledged that an older adolescent may prefer guidance sessions without their parents to allow them to experience a sense of independence and responsibility for their own lives: *“Perhaps for older adolescents*,* I think they really benefit from having that space […]*,* feeling as though they’re an individual that has*,* you know*,* some onus over their own life and what they choose*,* or choose not to do” (HCP2).* One young person, *CYP10*, explained how guidance sessions without a parent present can foster an environment that encourages honesty and openness: *“It can be hard to open up in front of your parents […] whereas if it’s just you and a therapist*,* you’re like ‘right yeah*,* you know*,* I’ll be honest’”.*

Participants highlighted that family dynamics and the strength of the parent-child relationship also play a crucial role in determining the extent of parental involvement in a self-help intervention: *“You can’t assume that all parents are going to be okay with the fact that their child has this disorder*,* and not all of them would be willing to help*,* and some might not react very well” (CYP2).*

### Person-centred approach: “The jigsaw pieces are here, but you can put the pieces together and you can make the picture that you want” (P9)

CYP and parents acknowledged that, despite variations in the underlying causes and presenting symptoms, there might be a common mechanism across all eating disorders – using eating disorder behaviours as a coping strategy: *“[Eating disorders] are all a result of children’s feelings and the fact that they can’t cope with those feelings*,* and they control it via food. So*,* I don’t suppose it really makes any difference whether they’re obviously eating loads and then being sick or whether they’re starving*,* it’s just a different approach… but it’s all to do with feelings” (P6).* One parent, *P3*, highlighted the overlapping nature of different eating disorder behaviours while sharing insights into her own child’s experiences: *“My daughter’s […] been diagnosed with anorexia*,* but she also binged at the beginning. Even though she’s been diagnosed with anorexia nervosa*,* she’s done all the others […]. I think that if you’re classed as a binge eater*,* there’s still some anorexia in there as well”*. Some CYP expressed that a transdiagnostic approach to guided self-help could be beneficial in reducing stigmatisation by removing rigid diagnostic labels and categories.

*CYP5: “I think [eating disorders] can all kind of be because of this […] root cause as a way to control*,* as a way to cope. And so*,* in that aspect they are very similar*,* even though people*,* you know*,* put different labels on them*,* put them into different sections*,* different categories. So*,* I think from that point of view*,* it kind of takes some of the labels away*,* it kind of stops people from putting them into different boxes being like ‘this one is more validated*,* this one’s less’”*.

However, there were reservations expressed by some participants about the practicality of implementing a broad approach. Concerns were raised that making the information too general might not effectively meet the specific needs of individuals: *“I think it’d be a bit of a nightmare to implement and I don’t know if it would necessarily meet anyone’s needs” (HCP1).*

In recognition of the heterogenous nature of eating disorders, all three stakeholder groups expressed the view that a guided self-help intervention should adopt a flexible and individualised approach that can be tailored to the CYP’s unique presentation: “*If there was a way to try and build a plan that was*,* you know*,* tailored to your child that would be great I think*,* because […] one thing works for one child and doesn’t for another” (P1).* There was a clear preference for a modular approach, wherein the guide works collaboratively with the family to determine the most suitable modules for the CYP based on their specific needs. Such an approach was seen as beneficial because it allows individuals to selectively engage with content that is more relevant to their own difficulties.

*CYP3: “I think it’d be good*,* because whilst lots of people do share symptoms*,* there’s loads of individualities and I guess if there was just that one guide*,* but then you could dip into different parts of it that are more relevant and less relevant to you*,* then I think it’d be good. Because I guess anxiety could be more prevalent in someone’s case whereas body image could be in another one’s case*,* and then um*,* they could just tailor it to have a more individualistic approach”*.

Participants described how giving CYP ownership of module selection could be empowering: *“Being able to pick out elements that resonate at that particular point gives you the power back” (P9).* However, some participants spoke about the risks of over tailoring resources to individuals and how this may result in them missing potentially valuable information. One young person, *CYP10*, described the potential benefit of exploring content that appears unrelated as it may unexpectedly resonate with their own experiences: *“Sometimes you find in the modules that you think don’t apply to you*,* you find things that make you go ‘oh*,* like maybe that is affecting me’. And so I think if you tailored it just to the individual*,* you might lose bits that […] they’d actually go ‘actually that’s*,* that’s quite helpful’”*.

### Diversity in intervention delivery: “It should be in any and every medium it can be to engage as many people as possible” (HCP4)

Stakeholders expressed a range of preferences for the proposed format of a guided self-help intervention, ranging from tangible resources like books to interactive apps and online platforms. CYP, in particular, placed importance on the concrete nature of a book and being able to write down thoughts and feelings on a physical page: “*Whether it’s a book or a booklet or something*,* but just something concrete. Um*,* I think it’s a lot easier to express yourself when actually writing something down rather than like clicking a button online” (CYP3).* This was corroborated by a healthcare professional *(HCP10)* who reflected on her experience of delivering self-help programmes: *“One of the things that they liked about the manual is being able to scribble and draw and write in it. The fact that it’s a […] physical copy*,* one of the others things is you can go back and add to it and reread it together in the sessions which a lot of young people liked”.*

However, some participants favoured the interactivity of an app or online platform, recognising its potential to provide engaging features such as videos, audio clips and online quizzes. One parent, *P10*, spoke about how an interactive app would enable users to access information in more manageable portions: *“In my head I can almost see like a kind of app*,* um*,* in it […] there might be something that’s almost like something interactive*,* bite-size chunks of information where you can go in and quickly pick and choose what to pull out*,* what’s relevant” (P10).*

The idea of delivering the intervention via an interactive PDF was proposed as a potential middle ground, combining the interactive features of an online platform with the ability to annotate and enter reflections: *“I quite like the idea of an editable PDF being sent out*,* because I think it’s a bit easier than going on to a website and finding it yourself and it’s sort of less intrusive than getting a booklet through*,* because I don’t have to sit down and open my booklet and go ‘right*,* okay we’re on page 80 today’. I can just sort of go ‘right*,* we’re on module four*,* here we go*,* here’s the 13 pages I’ve got’ and it’s sort of there and on email every week. I think that’s really helpful because it doesn’t feel lumber some*,* but it’s there and I can access it really easily” (CYP10).*

Stakeholders recognised the advantages and drawbacks of different mediums for intervention materials, and suggested that the intervention be offered in multiple formats to allow CYP to choose one that aligns with their personal preferences: *“It should have different media to access different people*,* I think so people can choose their preferred way” (P11).*

While the stakeholders demonstrated a clear preference for guided self-help over pure-self help, there was a lack of consensus regarding the specifics of how this guidance should be delivered, including session frequency, duration and format. Suggestions for session frequency varied, ranging from three sessions at key programme points, weekly sessions, to guidance on demand. Stakeholders emphasised the importance of considering patient preference: *“Have the young person choose*,* if they need that extra support they can have it” (CYP1).* Similarly, stakeholders expressed differing views on face-to-face versus remote guidance, recognising challenges in both approaches: *“I feel like obviously with remote*,* it’s harder to obviously see the person and almost how they appear*,* so I feel like it could be detrimental*,* and it is a fine line. Equally I feel like remote […] is easier to implement” (CYP11).* There was, however, agreement that if delivered remotely, guidance sessions should be over videocalls to enhance the connection between the CYP and guide: *“Over a Zoom or a Teams meeting*,* it’s nice to see someone” (P11).*

Stakeholders expressed concerns about the lack of diversity in existing materials related to eating disorders. There was a perception that these resources often portray a specific demographic, typically a young, white, middle-class female from a nuclear family. The importance of avoiding gender-specific assumptions was highlighted, urging the use of gender-neutral language and visuals to make resources more accessible and relevant to a diverse audience: *“I suppose it’s the […] non-gendered language and things like that and actually the use of pictures or colours or things […] that actually feel more neutral as opposed to gender specific” (HCP4).* Healthcare professionals also emphasised the need for cultural sensitivity in self-help materials: “*Eating disorders can present differently across different cultures […]*,* so I guess that being something to consider in terms of like examples that are being used to how people might relate to the material” (HCP9).*

## Discussion

### Main findings

The current study highlighted three themes central to key stakeholders’ perspectives on the use of guided self-help interventions for CYP with eating disorders: the role of guided self-help in bridging the treatment gap, the acceptability of guided self-help for this population and the need for a personalised and flexible approach.

CYP and parents had insight into their unmet treatment needs within the current service provision for eating disorders. Consistent with previous literature [25, 30], stakeholders noted advantages of guided self-help in its ability to increase access to psychological support and overcome long waiting times. This is especially crucial given that many CYP with eating disorders are waiting several weeks or months for specialist intervention [6]. Existing treatments were criticised for being centred on physical recovery and insufficient focus on psychological wellbeing, echoing wider literature on family based treatments that suggest adolescents may perceive FBT for anorexia nervosa as ‘too symptom focused’ [40]. Guided self-help, in contrast, was viewed positively for its emphasis on psychological aspects; enhancing knowledge, skills and coping strategies for CYP to manage their thoughts and feelings.

Stakeholders acknowledged that guided self-help interventions necessitate a certain level of commitment and motivation, and the most frequently cited determinant of CYP’s suitability for guided self-help was ‘level of readiness’ for change. This is not a novel finding; several studies have identified motivation and readiness to change as a significant predictor of treatment outcome for individuals with eating disorders, particularly in anorexia nervosa (see Vall & Wade [41] for meta-analysis). Nevertheless, the current findings affirm the central role of the guide in promoting CYP’s motivation to engage with guided self-help [24, 42, 43]. Participants also emphasised the critical importance of weight restoration for individuals who are severely underweight to have the cognitive capacity to engage with and derive benefit from guided self-help, a perspective that extends to psychological therapies more broadly [44]. As noted by Troscianko & Leon (2020), “psychological therapeutic work in a malnourished state is rarely a feasible option” [45]. Furthermore, it was agreed that guided self-help interventions are not suitable for CYP who present with high risk (e.g., extremely low weight, rapid weight loss, extreme low mood) which is in concert with the criteria outlined in a recent expert consensus statement from the UK [12].

Guided self-help interventions have traditionally been positioned as either the ‘first’ or ‘last step’ in a stepped-care model – serving as an early intervention [46] or a tool for relapse prevention/aftercare [47, 48]. However, emerging literature is starting to recognise that self-help can be administered at various points along the care pathway, including prevention/early intervention, augmentation of specialist intervention, relapse prevention/aftercare support, and as a stand-alone treatment [49, 50]. The present study highlights that the optimal point of guided self-help interventions on the care pathway for CYP with eating disorders remains a subject of ongoing debate. Some participants suggested that guided self-help should be offered as a stand-alone, low intensity psychological intervention, whereas others felt it served better as an adjunct to specialist treatment. Nevertheless, the potential for guided self-help to address gaps in existing service provision by providing psychoeducation, coping strategies, and opportunities for individualised self-reflection was emphasised. There was also consensus that, once proven in efficacy, guided self-help should be portrayed as an effective intervention to enhance its acceptability as an approach, not as ‘second best’ to therapist input and irrespective of its placement in the care pathway. This finding is akin to the framing effect described by Glare and colleagues [51].

Eating disorders have heterogeneous presentations, and individuals often migrate from one diagnosis to another [52]. They are also highly comorbid with other psychiatric disorders [53] which can complicate treatment efforts [54]. It was, therefore, not surprising, that the diverse nature of eating disorders was identified as a subtheme. Co-occurring conditions were prevalent among CYP in this study, and all stakeholder groups underscored the importance of future guided self-help interventions accounting for these comorbidities. Importantly, Wade and colleagues [55] have recently proposed a four-step protocol to adopt in the face of co-occurring mental health conditions in eating disorder treatments that can be applied to guided self-help.

Stakeholders also recognised commonalities in CYP with eating disorders, acknowledging that eating disorder behaviours often serve as auxiliary coping mechanisms: helping individuals manage emotional states and feel a sense of control over their eating, shape and weight concerns. A transdiagnostic approach that cuts across diagnostic boundaries was viewed as a means to address this underlying mechanism, as well as the diagnostic migration experienced by many CYP with eating disorders [56].

The primary rationale for transdiagnostic interventions lies in their potential to effectively address the heterogeneity and comorbidity that is the modal presentation in real-life settings [57]. However, a drawback of some transdiagnostic interventions is that their universality precludes tailored selection of treatment elements to the unique presentations of individuals [58]. This was reflected in the current findings; stakeholders expressed concerns that a universal approach to guided self-help interventions may not adequately meet the individual needs of CYP with eating disorders. As a solution, they advocated for a transdiagnostic intervention that was flexible and modular in approach, allowing for module selection and order to be tailored to specific needs and preferences of each young person. Such an approach has shown promise in other CYP populations (e.g., 53).

Stakeholders unanimously agreed that self-help materials should be age-appropriate and inclusive of all backgrounds, containing evidence-based and certified content to instil trust among users. Psychological interventions for CYP with mental health difficulties are often downward adaptations of adult treatments [59], such as the one Schmidt and colleagues [15] used in their study of adolescents with bulimia nervosa. The current findings suggest that innovations in the design and content of guided self-help interventions must carefully consider the specific developmental needs of CYP with eating disorders. Consistent with past studies [24, 43], the importance of a guide was emphasised; to help clarify challenging concepts, encourage skills practice, troubleshoot barriers to change and increase motivation. Parents were also seen as a potential facilitator to support the delivery of guided self-help, particularly for younger children. However, beyond these considerations, there were no clear preferences regarding the format of intervention delivery (e.g., book vs. online platform) or guidance type (i.e., session frequency, duration and format).

### Strengths and limitations

A major strength of the study is that it incorporated the views and opinions of three key stakeholder groups – CYP, parents and healthcare professionals – offering valuable insights into the potential utility of guided self-help interventions for CYP with eating disorders. Another strength lies in the inclusion of CYP with anorexia nervosa. Prior research has tended to exclude individuals with anorexia nervosa [29, 30], perhaps due to cautiousness regarding the use of self-help among individuals with higher medical risks [10]. The decision to recruit CYP with anorexia nervosa in this study was made so to not pre-determine suitability of guided self-help based on diagnostic category alone. Similarly, the decision to recruit via community settings was made to reach CYP who do not access services. However, it is noteworthy that all CYP who participated in the current study reported having current, or historic, treatment with specialist eating disorder services.

It is important to acknowledge that the current findings may not wholly represent the views of all CYP with eating disorders, nor parents or healthcare professionals, given the small and self-selected nature of the sample. While participants were geographically diverse, spanning various regions of England and including one parent from Scotland, the lack of demographic diversity is notable, primarily comprising White British females. No children under the age of 13 were recruited, possibly due to their limited presence on social media. Additionally, no fathers were included in the parent sample. Due to data limitations, socioeconomic status could not be determined. A limited range of eating disorders were also represented. Anorexia nervosa was the predominant diagnosis among CYP, and the majority of parents were parents of CYP with anorexia nervosa, which may be an artefact of the help-seeking population of CYP with eating disorders [60, 61]. Consequently, the themes presented here may be more reflective of CYP with anorexia nervosa, or, at minimum, CYP with restrictive eating disorders. Future research should explore more diverse recruitment strategies to capture a broader range of perspectives on the potential utility of guided self-help interventions for CYP with eating disorders.

There are three additional limitations to acknowledge. Firstly, it is unrealistic to entirely remove the research team’s prior knowledge and epistemological position in data collection and analysis. The lead researcher (*removed for peer review*) conducted this study as part of her doctoral research that aims to increase access to psychological treatment for CYP with eating disorders. The wider research team have considerable knowledge and clinical experience in treatments for CYP with eating disorders, each with their own therapeutic orientation. This inherent knowledge may have introduced bias into both data collection and interpretation. It is plausible that researchers aligned with FBT, for example, may have offered alternative interpretations. Relatedly, member checking, which involves participants being re-contacted to provide their views on interpretation, analyses and drafts [62], was not employed due to constraints in time and resources associated with the project. Lastly, several identified themes extend beyond the scope of guided self-help interventions to encompass psychological interventions more broadly, especially the third theme, ‘One size does not fit all’.

### Implications

The current findings have clear practical implications for the future design and delivery of guided self-help interventions for CYP with eating disorders. Guided self-help was perceived as a viable option to bridge the existing treatment gap, providing more accessible psychological support to CYP with eating disorders. Research suggests that psychological interventions with low acceptability may not be optimally delivered by providers or well-utilised by patients [20]. Thus, to improve the acceptability and increase the likelihood of subsequent adoption, guided self-help interventions should be developed and positioned as an avenue to empower CYP to enhance their knowledge of eating disorders and to acquire skills and coping strategies to manage their difficulties. A flexible approach is imperative – one that allows for individual choice and tailoring to individual needs and preferences. Future developments must also consider the specific developmental needs of CYP with eating disorders, and the role of parents in facilitating intervention delivery.

Although not covered here, stakeholders proposed specific content and design features to incorporate into future guided self-help interventions for CYP with eating disorders. However, clinical expertise and patient values are only two legs of the ‘three legged-stool’ of evidence-based practice [63]. All three components of evidence-based practice – research evidence, clinical expertise, and patient values – are integral for the optimal treatment of eating disorders [64]. Future work must integrate stakeholder recommendations for content and features, with the best available research evidence, to optimise the development of guided self-help interventions for CYP with eating disorders.

## Conclusion

The current study lays a foundation for the future design and delivery of guided self-help interventions for CYP with eating disorders. It provides important insights into key aspects of guided self-help that can enhance the acceptability and increase the likelihood of subsequent adoption – notably, the need to tailor the intervention materials and delivery to the individual needs and preferences of CYP with eating disorders. Notwithstanding the study limitations, the findings suggest that stakeholders perceive guided self-help interventions for CYP with eating disorders as an acceptable treatment approach that holds promise in addressing the increasing demand for psychological support among this patient group.

## Electronic supplementary material

Below is the link to the electronic supplementary material.

Supplementary Material 1

Supplementary Material 2

Supplementary Material 3

## Data Availability

No datasets were generated or analysed during the current study.

## Abbreviations

CYP: Children and Young People
HCP: Healthcare Professional
OSFED: Other Specified Feeding or Eating Disorder
ARFID: Avoidant/Restrictive Food Intake Disorder
