# Supplementary material for: “The only way that they can access help quickly”: a qualitative exploration of key stakeholders’ perspectives on guided self-help interventions for children and young people with eating disorders
Source: J Eat Disord. 2024 Sep 30;12:149. doi: 10.1186/s40337-024-01113-w (PMC11441004; doi:10.1186/s40337-024-01113-w)
Supplement: Supplementary file 1 — Supplementary Material 1 [file 40337_2024_1113_MOESM1_ESM.docx]

**Consolidated criteria for Reporting Qualitative Research (COREQ) checklist**

| **Topic** | **Item No.** | **Guide Questions/Description** | **Reported on Page No.** |
| --- | --- | --- | --- |
| **Domain 1: Research team and reflexivity** | | | |
| *Personal characteristics* | | | |
| Interview/facilitator | 1 | Which author/s conducted the interview or focus group? | 8 |
| Credentials | 2 | What were the researcher’s credentials? E.g., PhD, MD | 31 |
| Occupation | 3 | What was their occupation at the time of the study? | 31 |
| Gender | 4 | Was the researcher male or female? | 31 |
| Experience and training | 5 | What experience or training did the researcher have? | 31 |
| *Relationship with participants* | | | |
| Relationship established | 6 | Was a relationship established prior to study commencement? | 8 |
| Participant knowledge of the interviewer | 7 | What did the participants know about the research? E.g., personal goals, reasons for doing the research | 8 |
| Interviewer characteristics | 8 | What characteristics were reporting about the interviewer/facilitator? E.g., bias, assumptions, reasons and interests in the research topic | 31 |
| **Domain 2: Study design** | | | |
| *Theoretical framework* | | | |
| Methodological orientation and theory | 9 | What methodological orientation was stated to underpin the study? e.g. grounded theory, discourse analysis, ethnography, phenomenology, content analysis | 10 |
| *Participant selection* | | | |
| Sampling | 10 | How were participants selected? e.g., purposive, convenience, consecutive, snowball | 8 |
| Method of approach | 11 | How were participants approached? e.g., face-to-face, telephone, mail, email | 8 |
| Sample size | 12 | How many participants were in the study? | 11 |
| Non-participation | 13 | How many people refused to participate or dropped out? Reasons? | N/A |
| *Setting* | | | |
| Setting of data collection | 14 | Where was the data collected? E.g., home, clinic, workplace | 9 |
| Presence of non-participants | 15 | Was anyone else present besides the participants and researchers? | N/A |
| Description of sample | 16 | What are the important characteristics of the sample? E.g., demographic data | 11 |
| *Data collection* | | | |
| Interview guide | 17 | Were questions, prompts, guides provided by the authors? Was it pilot tested? | 9 |
| Repeat interviews | 18 | Were repeat interviews carried out? If yes, how many? | N/A |
| Audio/visual recording | 19 | Did the research use audio or visual recording to collect the data? | 9 |
| Field notes | 20 | Were field notes made during and/or after the interview or focus group? | 9 |
| Duration | 21 | What was the durations of the interviews or focus group? | 9 |
| Data saturation | 22 | Was data saturation discussed? | 9 |
| Transcripts returned | 23 | Were transcripts returned to participants for comment and/or correction? | 31 |
| **Domain 3: Analysis and findings** | | | |
| *Data analysis* | | | |
| Number of data coders | 24 | How many data coders coded the data? | 10 |
| Description of the coding tree | 25 | Did authors provide a description of the coding tree? | N/A |
| Derivation of themes | 26 | Were themes identified in advance or derived from the data? | 10 |
| Participant checking | 28 | Did participants provide feedback on the findings? | 31 |
| *Reporting* | | | |
| Quotations presented | 29 | Were participant quotations presented to illustrate the themes/findings? Was each quotation identified? E.g., participant number | 14-26 |
| Data and findings consistent | 30 | Was there consistency between the data presented and the findings? | 14-26 |
| Clarity of major themes | 31 | Were major themes clearly presented in the findings? | 14-26 |
| Clarity of minor themes | 32 | Is there a description of diverse cases or discussion of minor themes? | 14-26 |
| Developed from: Tong, A., Sainsbury, P., & Craig, J. (2007). Consolidated criteria for  reporting qualitative research (COREQ): a 32-item checklist for interviews and focus groups. *International Journal for Quality in Health Care*, 19(6), 349-357. | | | |
