# Supplementary material for: “The only way that they can access help quickly”: a qualitative exploration of key stakeholders’ perspectives on guided self-help interventions for children and young people with eating disorders
Source: J Eat Disord. 2024 Sep 30;12:149. doi: 10.1186/s40337-024-01113-w (PMC11441004; doi:10.1186/s40337-024-01113-w)
Supplement: Supplementary file 2 — Supplementary Material 2 [file 40337_2024_1113_MOESM2_ESM.docx]

Example Topic Guide for Children and Parents

**Past experience of self-help**

- What resources have you used to help manage your/your child’s eating disorder?
- *Probe:* Have you used any books? Websites? Apps? apps?
- What did you find helpful/unhelpful about these resources?

**Views on self-help for patient group**

- What do you think about a self-help programme for children and young people with eating disorders?
- *Probe:* Would you/your child use a self-help programme?
- What would be helpful/unhelpful about a self-help programme?

**Views on access**

- Is there a time when a self-help programme would be most useful to you/your child?
- Through what avenues should people be able to access the self-help programme?

**Views on format**

- What do you think a self-help programme should look like?
- *Probe:* Do you have any thoughts on the format of a self-help programme?
- *Probe:* Do you have any thoughts on whether it should be online or in book form?
- How long do you think the programme should be?

**Views on content**

- What are the key areas that a self-help programme should address?
- *Probe:* What would be helpful/unhelpful to include in the programme?
- *Probe:* What components does everyone need to have?

**Views on therapist involvement**

- Do you have any thoughts on whether someone should support the self-help programme (i.e., guided self-help)?
- What should this guidance look like?
- *Probe:* Should this guidance by over the phone, video or face-to-face?
- Do you have any thoughts on who should support the self-help programme?

**Views on parental involvement**

- Do you think parents be involved in the self-help programme?
- *Probe:* To what extent should parents be involved?
- *Probe:* Are there some cases where parents should/should not be involved?
- *Probe:* Why would that be helpful or not helpful?

Example Topic Guide for Healthcare Professionals

**Past experience of self-help**

- Have you got any experience of using self-help programmes in your clinical work?
- Where do you signpost your patients for more information?

**Views on self-help for patient group**

- What do you think about a self-help programme for children and young people with eating disorders?
- *Probe:* Do you think a self-help programme is suitable for children and young people with eating disorders?
- What would be helpful/unhelpful about a self-help programme?

**Views on access**

- Is there a time when a self-help programme would be most useful to a young person with an eating disorder?
- Through what avenues should people be able to access the self-help programme?

**Views on format**

- What do you think a self-help programme should look like?
- *Probe:* Do you have any thoughts on the format of a self-help programme?
- *Probe:* Do you have any thoughts on whether it should be online or in book form?
- How long do you think the programme should be?

**Views on content**

- What are the key areas that a self-help programme should address?
- *Probe:* What would be helpful/unhelpful to include in the programme?
- *Probe:* What components does everyone need to have?

**Views on therapist involvement**

- What are your thoughts on guided self-help vs pure self-help?
- What should guidance in guided self-help look like?
- *Probe:* Should this guidance by over the phone, video or face-to-face?
- Do you have any thoughts on who should support the self-help programme?

**Views on parental involvement**

- What are your views on parents/carers being involved in the self-help programme?
- *Probe:* To what extent should parents be involved?
- *Probe:* Are there some cases where parents should/should not be involved?
- *Probe:* Why would that be helpful or not helpful?

**Views on how to address the diversity and inclusion agenda**

- Should there be adaptations to self-help to address the diversity and inclusion agenda?
- *Probe:* What should these adaptations be?
