# Supplementary material for: “The only way that they can access help quickly”: a qualitative exploration of key stakeholders’ perspectives on guided self-help interventions for children and young people with eating disorders
Source: J Eat Disord. 2024 Sep 30;12:149. doi: 10.1186/s40337-024-01113-w (PMC11441004; doi:10.1186/s40337-024-01113-w)
Supplement: Supplementary file 3 — Supplementary Material 3 [file 40337_2024_1113_MOESM3_ESM.docx]

| **Table 1** |  |  |  |  |  |  |  |
| --- | --- | --- | --- | --- | --- | --- | --- |
| *Individual participant characteristics of CYP sample* | | | | | | | |
| Participant ID | FG/I | Age | Gender | Ethnicity | ED diagnosis^a^ | ED treatment^b^ |  |
| CYP1 | FG1 | 13 | Female | White British | AN | Current; NHS |  |
| CYP2 | FG2 | 17 | Female | Any other Asian background | AN | Current; NHS |  |
| CYP3 | FG2 | 16 | Female | White British | AN | Current; NHS |  |
| CYP4 | FG2 | 17 | Female | White British | OSFED | Past; NHS |  |
| CYP5 | FG2 | 16 | Female | White British | AN | Current; NHS & Private |  |
| CYP6 | FG1 | 14 | Female | White British | AN | Current; NHS |  |
| CYP7 | FG1 | 13 | Female | White British | AN | Current; NHS |  |
| CYP8 | FG1 | 14 | Female | White British | OSFED | Current; NHS |  |
| CYP9 | I3 | 13 | Female | White British | AN | Current; NHS |  |
| CYP10 | I1 | 19 | Male | White British | AN | Current; Third sector |  |
| CYP11 | I2 | 18 | Female | White British | AN & BN | Past; NHS & Third sector |  |
| CYP = Children and Young People; FG = Focus Group; I = Interview; ED = Eating Disorder; AN = Anorexia Nervosa; OSFED = Other Specified Feeding or Eating Disorder; BN = Bulimia Nervosa  ^a^Self-reported lifetime eating disorder diagnosis; ^b^Experience of eating disorder treatment | | | | | | | |

| **Table 2** | | | | | | | | | | | | | |
| --- | --- | --- | --- | --- | --- | --- | --- | --- | --- | --- | --- | --- | --- |
| *Individual participant characteristics of parent sample* | | | | | | | | | | | | | |
| Parent | | | | | |  | Child | | | | | | |
| Participant ID | FG/I | Caregiver | Age | Gender | Ethnicity |  | Participant ID^a^ | Age | Gender | Ethnicity | ED diagnosis^b^ | ED treatment^c^ |  |
| P1 | FG2 | Mother | 52 | Female | White British |  | N/A | 15 | Female | White British | AN | Current; NHS |  |
| P2 | FG1 | Mother | 55 | Female | White British |  | CYP3 | 16 | Female | White British | AN | Current; NHS |  |
| P3 | FG1 | Mother | 45 | Female | White British |  | CYP1 | 13 | Female | White British | AN | Current; NHS |  |
| P4 | FG1 | Mother | 51 | Female | Any other White background |  | N/A | 16 | Male | White British | AN | Current; NHS |  |
| P5 | FG1 | Mother | 46 | Female | White British |  | CYP5 | 16 | Female | White British | AN | Current; NHS & Private |  |
| P6 | FG2 | Mother | 45 | Female | White British |  | CYP7 | 13 | Female | White British | AN | Current; NHS |  |
| P7 | FG2 | Mother | 48 | Female | White British |  | CYP8 | 14 | Female | White British | OSFED | Current; NHS |  |
| P8 | FG7 | Mother | 47 | Female | White British |  | CYP9 | 13 | Female | White British | AN | Current; NHS |  |
| P9 | FG7 | Mother | 55 | Female | White British |  | N/A | 17 | Female | White British | OSFED | Past; NHS & Private |  |
| P10 | FG2 | Mother | 42 | Female | White British |  | N/A | 12 | Female | White British | AN | Current; NHS |  |
| P11 | I4 | Mother | 53 | Female | Any other Asian background |  | N/A | 17 | Female | Any other Asian background | AN | Current; NHS |  |
| P12 | FG7 | Mother | 52 | Female | White British |  | N/A | 16 | Female | White and Black African | OSFED | Current; NHS |  |
| CYP = Children and Young People; FG = Focus Group; I = Interview; ED = Eating Disorder; AN = Anorexia Nervosa; OSFED = Other Specified Feeding or Eating Disorder  ^a^Participant ID of participating child in study; ^b^Self-reported lifetime eating disorder diagnosis; ^c^Experience of eating disorder treatment | | | | | | | | | | | | | |

| **Table 3** |  |  |  |  |  |  |
| --- | --- | --- | --- | --- | --- | --- |
| *Individual participant characteristics of healthcare professional sample* | | | | | | |
| Participant ID | FG/I | Age | Gender | Ethnicity | Occupation | CYP ED experience^a^ |
| HCP1 | FG6 | 43 | Female | White British | Clinical Psychologist | 16 years |
| HCP2 | FG6 | 33 | Female | White British | Clinical Psychologist | 2 months |
| HCP3 | FG5 | 45 | Male | White Irish | Consultant Psychiatrist | 7 years 11 months |
| HCP4 | FG5 | 39 | Female | White British | RMN & CBT Therapist | 13 years |
| HCP5 | FG5 | 52 | Female | White British | RMN | 12 years |
| HCP6 | FG6 | 42 | Male | White British | RMN & Family Therapist | 7 years |
| HCP7 | FG5 | 24 | Female | White British | Assistant Psychologist | 3 years |
| HCP8 | FG5 | 33 | Female | White British | Clinical Support Worker | 1 year 4 months |
| HCP9 | FG5 | 32 | Female | White British | Clinical Psychologist | 1 year 9 months |
| HCP10 | FG6 | Preferred not to say | Preferred not to say | White British | Clinical Psychologist | 25+ years |
| CYP = Children and Young People; HCP = Healthcare Professional; FG = Focus Group; I = Interview; ED = Eating Disorder; RMN = Mental Health Nurse; CBT = Cognitive Behavioural Therapy  ^a^Years of experience treating CYP with eating disorders | | | | | | |
|  |  |  |  |  |  |  |
